# Deletion of the NSm Virulence Gene of Rift Valley Fever Virus Inhibits Virus Replication in and Dissemination from the Midgut of *Aedes aegypti* Mosquitoes

**DOI:** 10.1371/journal.pntd.0002670

**Published:** 2014-02-13

**Authors:** Rebekah C. Kading, Mary B. Crabtree, Brian H. Bird, Stuart T. Nichol, Bobbie Rae Erickson, Kalanthe Horiuchi, Brad J. Biggerstaff, Barry R. Miller

**Affiliations:** 1 Division of Vector-Borne Diseases, Centers for Disease Control and Prevention, Fort Collins, Colorado, United States of America; 2 Viral Special Pathogens Branch, Division of High-Consequence Pathogens and Pathology, Centers for Disease Control and Prevention, Atlanta, Georgia, United States of America; United States Army Medical Research Institute of Infectious Diseases, United States of America

## Abstract

**Background:**

Previously, we investigated the role of the Rift Valley fever virus (RVFV) virulence genes NSs and NSm in mosquitoes and demonstrated that deletion of NSm significantly reduced the infection, dissemination, and transmission rates of RVFV in *Aedes aegypti* mosquitoes. The specific aim of this study was to further characterize midgut infection and escape barriers of RVFV in *Ae. aegypti* infected with reverse genetics-generated wild type RVFV (rRVF-wt) or RVFV lacking the NSm virulence gene (rRVF-ΔNSm) by examining sagittal sections of infected mosquitoes for viral antigen at various time points post-infection.

**Methodology and Principal Findings:**

*Ae. aegypti* mosquitoes were fed an infectious blood meal containing either rRVF-wt or rRVF-ΔNSm. On days 0, 1, 2, 3, 4, 6, 8, 10, 12, and 14 post-infection, mosquitoes from each experimental group were fixed in 4% paraformaldehyde, paraffin-embedded, sectioned, and examined for RVFV antigen by immunofluorescence assay. Remaining mosquitoes at day 14 were assayed for infection, dissemination, and transmission. Disseminated infections were observed in mosquitoes as early as three days post infection for both virus strains. However, infection rates for rRVF-ΔNSm were statistically significantly less than for rRVF-wt. Posterior midgut infections in mosquitoes infected with rRVF-wt were extensive, whereas midgut infections of mosquitoes infected with rRVF-ΔNSm were confined to one or a few small foci.

**Conclusions/Significance:**

Deletion of NSm resulted in the reduced ability of RVFV to enter, replicate, and disseminate from the midgut epithelial cells. NSm appears to have a functional role in the vector competence of mosquitoes for RVFV at the level of the midgut barrier.

## Author Summary

Rift Valley fever virus (RVFV) is a mosquito-borne virus endemic to Africa. Outbreaks of RVFV have resulted in devastating morbidity and mortality in livestock and humans. A novel RVFV vaccine strain has been developed in which two virulence genes, NSs and NSm, have been deleted from the RVFV genome. Previously, we demonstrated that deletion of NSm also significantly reduced the ability of *Aedes aegypti* mosquitoes to transmit RVFV. The objective of this study was to track the spread (dissemination) of wild type RVFV (rRVF-wt) and RVFV lacking the NSm virulence gene (rRVF-ΔNSm) through different tissues in the mosquito body over time by staining lengthwise slices of infected mosquitoes with fluorescent antibody specific to RVFV. We found that midgut infections in mosquitoes exposed to rRVF-wt were extensive, whereas midgut infections in mosquitoes infected with rRVF-ΔNSm were confined to only one or a few small foci. Our data provide supporting evidence that the NSm virulence gene has a functional role in mosquitoes by helping RVFV establish an infection in, and escape from, the midgut.

## Introduction

Rift Valley fever virus (RVFV) (family *Bunyaviridae*, genus *Phlebovirus*) is a zoonotic, mosquito-borne virus endemic to Africa. Human illness is typically febrile, but 1–2% of cases develop more severe disease including hepatitis, encephalitis, retinitis, vision loss, jaundice, severe anemia, neurologic manifestations, renal failure, and hemorrhagic fever [1]–[3]. A hallmark of RVFV outbreaks are “abortion storms” among sheep and cattle, with devastating mortality rates in newborn and young animals [4]–[5]. Transmission of RVFV is mosquito-borne. RVFV is registered as a Category A overlap select agent with both the U.S. Department of Agriculture Animal and Plant Health Inspection Service and the Centers for Disease Control and Prevention due to its biothreat potential and ability to cause significant economic losses to the livestock industry as well as substantial human morbidity and mortality [6]–[7]. The recent spread of RVFV to the Arabian Peninsula [8]–[9] serves as a precedent for the potential for this virus to be introduced into the United States or Europe, and highlights the need for preparedness and development of a safe and efficacious human and veterinary vaccine.

The RVFV genome is tripartite, negative sense RNA segments. Of the three segments, the small (S) segment codes for the nucleoprotein and the nonstructural NSs protein, the medium (M) segment encodes the two structural glycoproteins, Gn and Gc, as well as two nonstructural proteins (NSm and NSm-Gn) and the large (L) segment codes for the viral RNA-dependent RNA polymerase. Nonstructural protein NSs is known to inhibit IFN-β, promote degradation of PKR, and suppress host transcription [10]–[12] while NSm is involved in suppression of virus-induced apoptosis [13]. Both NSs and NSm are recognized to function as virulence factors, however, neither NSs nor NSm are individually required in cell culture for efficient virus replication, assembly, or maturation [13]–[16].

A number of vaccines have been developed against RVFV. However these vaccines have been plagued with many problems including poor immunogenicity, difficulties in manufacturing, and post-vaccination abortions and teratogenesis in livestock [4], [17]–[18]). In response to these problems, Bird et al. [19]–[20] developed a novel vaccine based on the deletion of NSs and NSm. Utilizing a reverse genetics system, infectious wild type and deletion mutant RVF viruses were reconstituted in cell culture from three plasmids encoding antigenomic copies of the S, M, and L segments; deletion mutant viruses lacked the NSs gene on the S segment, and/or the NSm gene on the M fragment [19]–[20]. The double deletion mutant RVFV was demonstrated to be protective and immunogenic in rats, mice, and sheep, without producing clinical illness in these animals [19]–[20]. Due to the enhanced safety profile of this vaccine candidate it was recently excluded from the Select Agent regulations and reclassified by the NIH Recombinant Advisory Committee and the CDC as requiring BSL-2 safety precautions [21]. Deletion of NSm alone retained some ability to cause lethal hepatic and neurologic disease in Wistar-Furth rats and has been developed as an animal model for human delayed onset encephalitic disease [22].

The non-essential nature of NSm for growth in vertebrate cell culture or to mammalian pathogenesis prompted investigations into the role of this protein in RVFV infection and replication in the mosquito vector [23]. Indeed, deletion of NSm greatly reduced the infection, dissemination, and transmission rates of RVFV in *Aedes aegypti* mosquitoes and infection rates in *Culex quinquefasciatus* mosquitoes [23]. The specific aim of this study was to further characterize midgut infection and escape barriers of RVFV in *Ae. aegypti* infected with reverse genetics-generated wild type RVFV (rRVF-wt) or RVFV lacking the NSm virulence gene (rRVF-ΔNSm) by examining sagittal sections of infected mosquitoes for viral antigen by immunofluorescence at various time points post-infection.

## Materials and Methods

### Mosquitoes and viruses

The *Ae. aegypti* Rexville D mosquito strain used was an isofemale line derived from a population of *Ae. aegypti* collected as larvae in San Juan, Puerto Rico (Rexville) in 1991 [24]. Mosquitoes were double-caged in screened paperboard pint containers inside environmental chambers at 28°C and approximately 95% relative humidity. Reverse genetics-generated viruses, rRVF-wt and rRVF-ÄNSm, were used in this study [19], [22].

### Mosquito infections

To maximize infectivity to mosquitoes, freshly-harvested rRVF-wt and rRVF-ÄNSm virus strains were used in the infectious blood meal. Three days prior to the infectious blood-feed, one T-75 flask each of Vero cells was inoculated with either rRVF-wt or rRVF-ÄNSm at a multiplicity of infection (MOI) of 0.1. On Day 3 post-infection, cell-culture supernatant was harvested and clarified for use in the infectious blood meal. Because differences in virus concentration may affect mosquito vector competence [23], we attempted to equalize the virus titers of rRVF-wt and rRVF-ΔNSm in the mosquito blood meals. RNA was extracted from clarified supernatant from flasks containing freshly-grown virus and quantified by qRT-PCR using novel primers and a probe targeting the polymerase gene: 4108F (5′-TTT AGA GAC CGT TTG AAC ATA CC-3′), 4217R (5′-GCA ATG CGC AAC AAT ATT TCT-3′) and probe, 4161P (5′FAM-TC CAG AGG TGC TCT ATC GGG CTC C-3′). The observed difference in RNA copies/mL between rRVF-wt and rRVF-ΔNSm were corrected by diluting the rRVF-wt virus supernatant in Dulbecco's modified Eagle's media by a factor of 2.8 prior to preparing the infectious blood meals.

The infectious blood meals were prepared by mixing two parts washed defibrinated calf blood with two parts virus and one part FBS+10% sucrose. A virus-negative blood meal contained cell culture media in place of virus-positive cell supernatant. Blood was warmed to 37°C in a water bath. Adult 8- to 10-day-old *Ae. aegypti* mosquitoes starved for 27-hours were administered an infectious RVFV blood meal containing either rRVF-wt or rRVF-ΔNSm on blood-soaked cotton balls. Screened pint cups containing 100–150 female *Ae. aegypti* were placed inside plastic bins inside a 28°C environmental chamber. One blood-soaked cotton ball was placed on each carton for 25 minutes. Five hundred microliters of each blood meal and 500 µl of virus seed brought to 20% FBS were frozen at −80°C for later quantification. Following the blood meal, mosquitoes were anesthetized by freezing at −20°C for 1 min, and fully engorged mosquitoes were sorted over ice inside of a glove box; only fully-engorged mosquitoes were used for the experiment. Engorged mosquitoes were placed into screened 3.8L paperboard cartons and supplied with 5% sugar solution. Paperboard cartons were placed inside a 30.5 cm^3^ metal cage inside the environmental chamber for double containment. On days 0, 1, 2, 3, 4, 6, 8, 10, 12, and 14 post-infection, between 10 and 16 mosquitoes from each experimental group were harvested for pathology and frozen at −80°C. Remaining mosquitoes at day 14 were processed for vector competence and analyzed statistically as described by Crabtree et al. [23]. Plaque titrations were performed on Vero cells as described by Miller et al. [25], with the second overlay on day three. All work involving manipulations with infectious virus or infected mosquitoes was performed in BSL3+ containment.

### Paraformaldehyde fixation and staining of paraffin-embedded mosquitoes

The complete protocol used for fixing and antibody staining of paraffin-embedded sections of mosquitoes is described in detail by Kading et al. [26]. Spot slides of rRVF-wt-infected and uninfected Vero cells, as well as sections of rRVF-wt- and rRVF-ΔNSm-infected and uninfected *Ae. aegypti* from various time points were tested simultaneously served as positive and negative controls [26]. Embedding and sectioning was performed by Colorado HistoPrep. Mosquitoes were arranged vertically, four per section, with two sections per slide and 10 slides per block. The methodology for the staining of control spot slides and head squashes was described previously [27]. Mouse anti-RVFV strain ZH501 hyperimmune ascetic fluid diluted 1∶2500 was used as a primary antibody for immunofluorescence assays on control spot slides and head squashes; a dilution of 1∶1600 was used on paraffin-embedded sections. Goat anti-mouse IgG-Alexa 488 (Invitrogen, Baltimore, MD) diluted 1∶2000 served as the secondary antibody conjugate.

### Dissemination index

To quantify and compare virus dissemination to different mosquito tissues over time, the dissemination index was employed [28]–[29]. The dissemination index is based on the infection status of particular tissues that are nearly always infected in specimens with a disseminated infection. The presence or absence of viral antigen was scored in the following six tissues and the number of antigen-positive tissues was divided by six to give the dissemination index: ommatidia; fat body in the head, thorax and abdomen; salivary glands; and thoracic ganglia. A dissemination index of 1.0 indicated that all examined tissues were positive, whereas an index of zero indicated that dissemination had not yet occurred. Scatterplots of dissemination indices were generated in Prism. Overlapping points were nudged horizontally for visibility.

## Results

### Mosquito infections

*Aedes aegypti* mosquitoes received an artificial blood meal containing either 7.6 log_10_ pfu/mL rRVF-wt or 7.9 log_10_ pfu/mL rRVF-ΔNSm. Mosquitoes were harvested for pathology daily for the first five days and subsequently every other day until 14 days following the infectious blood meal (Table 1). Up to 12 mosquitoes per time point for each virus were submitted for paraffin-embedding and sectioning. On day 14, infection, dissemination and transmission rates were assayed for the remaining 25 mosquitoes infected with rRVF-wt and 30 mosquitoes infected with rRVF-ΔNSm (Table 2). Of the 21/25 mosquitoes remaining infected with rRVF-wt 14 days post-infection, the average titer was 5.5±0.30 log_10_ pfu. In contrast, 7/30 mosquitoes exposed to rRVF-ΔNSm were infected 14 days after the infectious blood meal; these mosquitoes had a titer of 2.87 log_10_ ±0.58 pfu. Accounting for virus uptake on day 0, these rates of sustained infection differed significantly (OR = 15.2, 95% CI 4.0–57.7). The difference in these log_10_ titers was 2.7 (95% CI 2.1–3.2), statistically significantly different from 0. Notably, the average titer in a mosquito exposed to rRVF-wt increased from 5.0 log_10_ pfu in the blood meal to 5.5 log_10_ pfu on day 14, whereas the average virus titer in a mosquito exposed to rRVF-ΔNSm dropped from 5.8 log_10_ pfu in the blood meal to 2.87 log_10_ pfu on day 14.

**Table 1:** Infection and dissemination rates of rRVF-wt and rRVF-ΔNSm in *Aedes aegypti* determined by immunofluorescence assay on sagittal sections of mosquitoes.

rRVF-wt										rRVF-ΔNSm								
Day	Infection Rate			Dissemination Rates						Infection Rate			Dissemination Rates					
				# pos/# exposed			# pos/# infected						# pos / # exposed			# pos/# infected		
	(n)	# pos	% pos	(n)	# pos	% pos	(n)	# pos	% pos	(n)	# pos	% pos	(n)	# pos	% pos	(n)	# pos	% pos
0	2	0	0	2	0	0	0	0	0	3	0	0	3	0	0	0	0	0
1	11	7	64	11	0	0	7	0	0	12	0	0	12	0	0	0	0	0
2	11	7	64	11	0	0	7	0	0	10	8	80	10	0	0	8	0	0
3	12	12	100	12	1	8	12	1	8	8	3	38	8	1	13	3	1	33
4	12	10	83	12	0	0	10	0	0	11	3	27	11	1	9	3	1	33
6	12	8	67	12	4	33	8	4	50	8	4	50	8	0	0	4	0	0
8	12	9	75	12	5	42	9	5	56	8	5	63	8	0	0	5	0	0
10	7	7	100	7	7	100	7	7	100	8	6	75	8	0	0	6	0	0
12	7	7	100	7	7	100	7	7	100	4	2	50	4	1	25	2	1	50
14	10	10	100	10	10	100	10	10	100	9	7	78	9	0	0	7	0	0

Mosquitoes on day 0 were harvested immediately after receiving an infectious blood meal and were not considered infected at the time of observation since no viral antigen was visible in the midgut epithelial cells.

**Table 2:** Infection, dissemination, and transmission rates of rRVF-wt and rRVF-ΔNSm in *Aedes aegypti* 14 days post infection.

Infection Rate^1^ *						Dissemination Rates				Transmission Rates			
						# pos/# exposed^2^ *		# pos/# infected^3^ *		# pos/# exposed^4^ *		# pos/# disseminated^5^ *	
Virus	Blood meal titer^6^	Avg (sd) Day 0 titer^7^	Avg (sd) Day 14 titer^8^	% pos	(n)	% pos	(n)	% pos	(n)	% pos	(n)	% pos	(n)
rRVF-wt	7.6	5.3 (0.04)	5.5 (0.3)	84.0	25	80.0	25	95.2	21	60.0	25	75.0	20
rRVF-ΔNSm	7.9	5.4 (0.17)	2.7 (0.58)	23.3	30	0.0	30	0.0	7	0.0	30	0.0	0

1 Number of mosquitoes containing detectable virus by plaque assay divided by the number of mosquitoes exposed to infectious blood meal.

2 D_e_ = number of mosquitoes with RVFV antigen in head tissues divided by number of mosquitoes exposed to infectious blood meal.

3 D_i_ = number of mosquitoes with RVFV antigen in head tissues divided by number of infected mosquitoes.

4 T_e_ = number of mosquitoes with RVFV-positive saliva by plaque assay divided by number of mosquitoes exposed to infectious blood meal.

5 T_d_ = number of mosquitoes with RVFV-positive saliva by plaque assay divided by number of mosquitoes with disseminated infection.

6 Titer expressed as log_10_ pfu/mL.

7 n = 3; titer expressed as log_10_ pfu/mosquito; average is the log_10_ geometric mean; sd = standard deviation.

8 rRVFV-wt n = 21; rRVFV-ΔNSm n = 7; titer expressed as log_10_ pfu/mosquito; average is the log_10_ geometric mean; sd = standard deviation.

* Rate difference between virus types is statistically significant.

### Pathology

**Figure 1:**
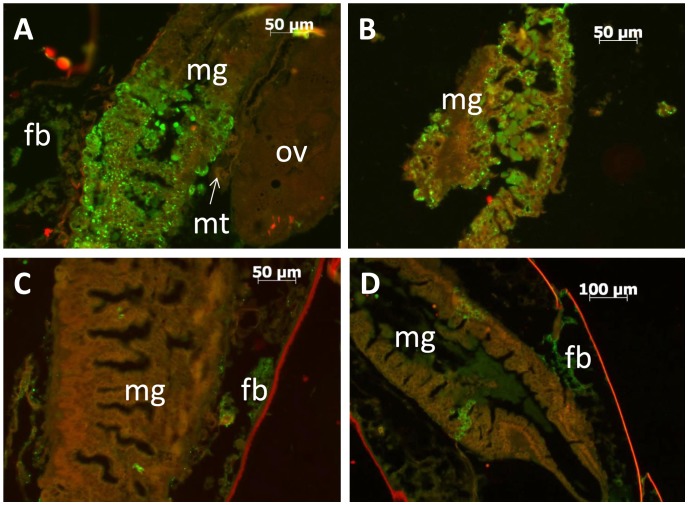
Replication of RVFV and dissemination of RVFV from in the posterior midgut is impaired in mosquitoes infected with rRVF-ΔNSm. A and B) Sagittal sections of two midguts of *Ae. aegypti* infected with rRVF-wt. C and D) Sagittal sections of two midguts of *Ae. aegypti* infected with rRVF-ΔNSm. Abbreviations: fb = fat body; mg = midgut; mt = Malpighian tubule; ov = ovary.

The dynamics of infection between rRVF-wt and rRVF-ΔNSm was distinctly different between the two virus strains. Posterior midgut infections in mosquitoes infected with rRVF-wt were extensive (Fig. 1 A and B) whereas infection of the posterior midgut in mosquitoes infected with rRVF-ΔNSm was confined to one or a few small foci (Fig. 1C and D). Disseminated infections were observed in mosquitoes by three days post infection for both viruses (Table 1). However, accounting for number of days post infection, infection rates for rRVF-ΔNSm were statistically significantly less than that of rRVF-wt (odds ratio (OR) = 0.20, 95% CI = 0.10–0.41) (Table 1). When modeling the dissemination rates, a statistically significant interaction was found between days post infection and virus type (OR = 0.53, 95% CI = 0.37–0.76). Since the dissemination rate of rRVF-wt increased with time but only three dissemination events were documented among mosquitoes infected with rRVF-ΔNSm, the difference between the two viruses also increased as days post infection increased. Similar results were found for the dissemination rates of only those mosquitoes that became infected (OR = 0.48, 95% CI = 0.32–0.72) (Table 1).

### Dissemination

**Figure 2:**
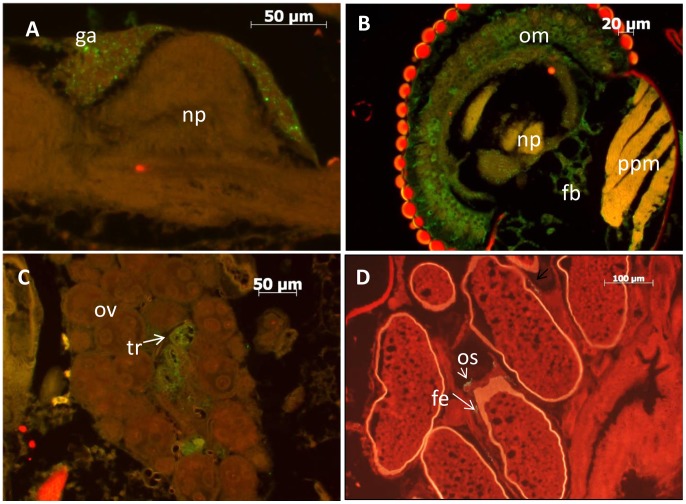
Infection of various *Aedes aegypti* tissues with rRVF-wt. A) ventral nerve cord, showing infection of the ganglia B) head, showing infection of the ommatidia and fat body, C) ovary, showing infection of the tracheal cells D) ovarioles, showing infection of the follicular epithelium and ovariole sheath. Abbreviations: fb = fat body; fe = follicular epithelium; ga = thoracic ganglia; np = neuropile; om = ommatidia; ov = ovary; os = ovariole sheath; ppm = pharyngeal pump musculature; tr = tracheoles.

**Figure 3:**
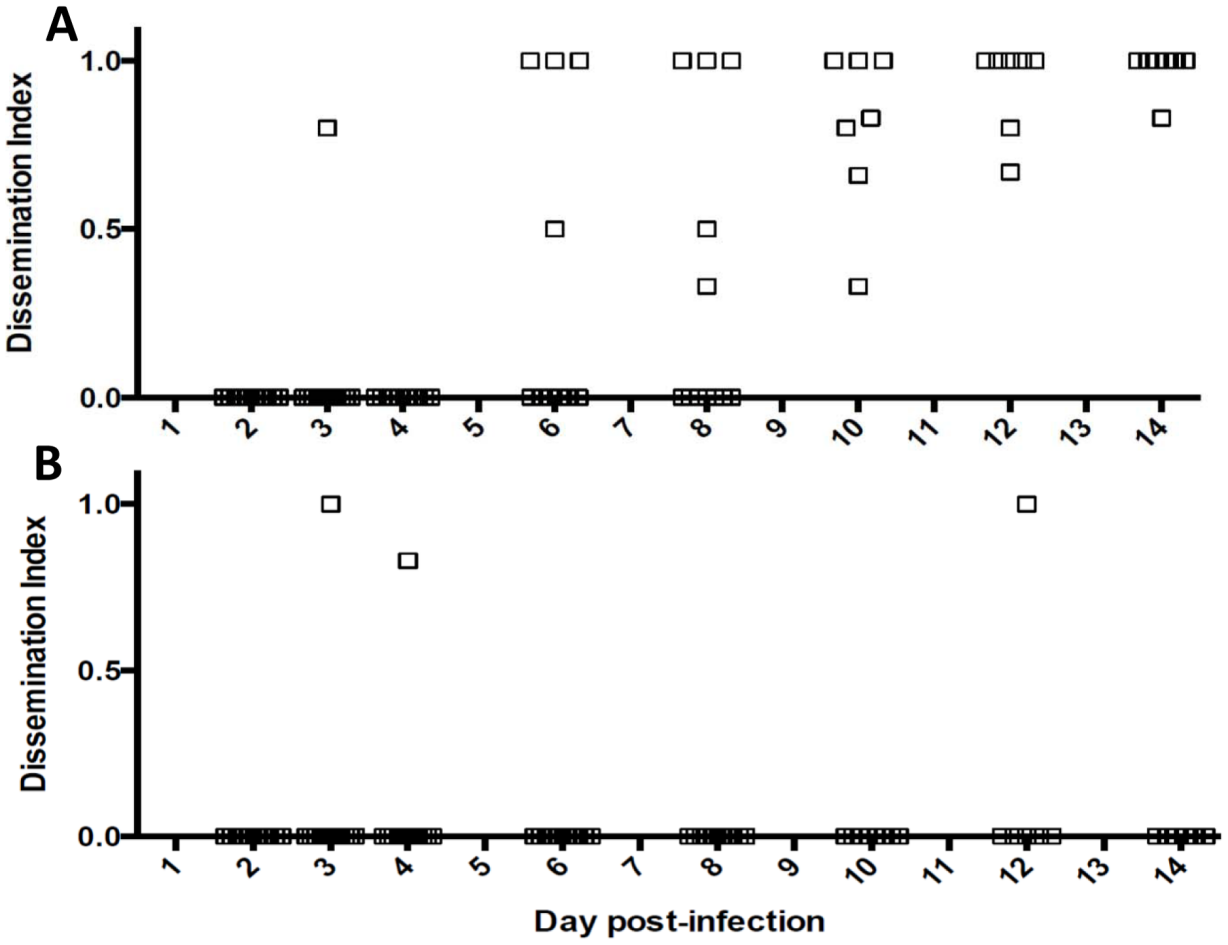
Dissemination indices of (A) rRVF-wt, and (B) rRVF-ΔNSm, in *Aedes aegypti*. Each dot represents one mosquito.

Other than the midgut, RVFV antigen was visible in a variety of tissues including the thoracic and abdominal fat body (Fig. 1A,C,D), thoracic ganglia (Fig. 2A), salivary glands (not shown), intussuscepted foregut (not shown), the ommatidia, fat body and nervous tissue of the head (Fig. 2B), tracheal cells of the ovaries (Fig. 2C), ovariole sheath and follicular epithelium (Fig. 2D), and in five specimens, the hindgut (not shown). RVFV antigen was not observed in the Malpighian tubules (Fig. 1A) or flight muscles. The time of dissemination varied from mosquito-to-mosquito but ranged between three to eight days post infection in mosquitoes exposed to rRVF-wt (Table 1, Fig. 3). Dissemination to other tissues was rapid for both rRVF-wt and rRVF-ΔNSm once the virus escaped from the midgut, as evidenced by the majority of specimens having a dissemination index of close to either zero, indicating no dissemination, or one, indicating all counted tissues were infected (Fig. 3).

### Vector competence

Infection, dissemination and transmission rates of *Ae. aegypti* exposed to rRVF-wt were all significantly higher than those of *Ae. aegypti* exposed to rRVF-ΔNSm (Table 2). None of the day 14 mosquitoes infected with rRVF-ΔNSm developed a disseminated infection or transmitted virus that was detectable by plaque titration.

## Discussion

Through transmission experiments and histological examination of infected mosquitoes, we have demonstrated that *Ae. aegypti* mosquitoes infected with RVFV lacking the NSm nonstructural protein gene have significantly lower infection, dissemination, and transmission rates than mosquitoes infected with wild-type RVFV [23, and this study] The barriers to infection in mosquitoes infected with rRVF-ΔNSm appear to be in the ability of the virus to replicate in and escape from epithelial cells of the posterior midgut. Small foci of RVFV antigen were visible in the midgut epithelial cells two days post infection for mosquitoes infected with the rRVF-ΔNSm deletion mutant virus. By day 14 post infection, rRVF-ΔNSm infection rates were not over 80%, and virus had disseminated from the midgut in only three mosquitoes (n = 81, 3.7%) (Table 1). We are confident that these three disseminations are not artifacts, as evidenced by the specific cell-associated staining depicted in Figure 1 and the clarity of our positive and negative controls (data not shown). In contrast, midgut infections in mosquitoes exposed to rRVF-wt were easily observable one day post-infection, and by histological examination of specimens, 100% infection and dissemination rates were observed on days 10, 12, and 14 post-infection (Table 1). An infection rate of 21/25 (84%) was also determined at day 14 by plaque assay for rRVF-wt-exposed mosquitoes in the transmission experiment (Table 2), for a combined day 14 infection rate of 31/35, or 88.6%. Replication of rRVF-ΔNSm was reduced to small foci in the midgut, compared to the extensive infections present in the midguts of mosquitoes infected with rRVF-wt (Fig. 1). Therefore, the absence of NSm resulted in a reduction in the ability of RVFV to establish an infection, and escape from the midgut.. These results are consistent with the findings of Crabtree et al. [23], who found that while infection and dissemination were severely inhibited by deletion of NSm, it was not blocked completely. In that study, a single mosquito (n = 129) exposed to rRVF-ΔNSm developed a disseminated infection and transmitted virus [23].

On a cellular level, it is not yet known how NSm promotes the ability of RVFV to establish an infection in and disseminate from the mosquito midgut. The biological function of NSm has remained largely obscure, as this protein is not necessary for viral growth in cell culture [14], [30], nor for pathogenesis in a mammalian host [22]. Won et al. [13] demonstrated that NSm suppressed virus-induced apoptosis through inhibiting STP-induced caspase 8 and caspase 9 activities. This antiapoptotic activity occurred in the absence of other viral proteins. More recently, Engdahl et al. [31] identified several murine proteins that interacted with the NSm protein of RVFV, providing additional clues regarding the role of NSm in RVFV infection. The strongest protein-protein interactions were found with the cleavage and polyadenylation specificity factor subunit 2 (Cpsf2) which functions in pre-mRNA 3′ end processing and formation, the peptidyl-prolyl cis-trans isomerase (cyclophilin)-like 2 protein (Ppil2) which has multiple functions spanning intracellular protein trafficking and regulation of chemotactic responses such as cell-mediated immunity and inflammation, and the 25 kDa synaptosome-associated protein (SNAP-25) which is involved in vesicle docking, membrane fusion and exocytosis, and Ca2^+^-dependent neurotransmission in neuronal cells of the brain [31]. It is unclear how these results translate to the role of NSm in the midgut of a mosquito vector. RNAi and autophagy have been recognized as anti-arboviral innate immune responses in insects [32]–[33]. The involvement of NSm in counteracting one or both of these processes seems reasonable, and warrants further investigation.

Our results regarding the timing of dissemination and distribution of RVFV antigen to various mosquito tissues are also consistent with those of previous studies of RVFV infections in mosquitoes. We observed virus dissemination from the posterior midgut by three days post infection for both rRVF-wt and rRVF-ΔNSm. Early dissemination has previously been reported for RVFV. Faran et al. [34] found that 6% of *Culex pipiens* L. infected with RVFV ZH-501 had a disseminated infection as early as 12 hours post infection, 9% had disseminated by 24 h and 22% had disseminated by 48 h. These results were confirmed by Romoser et al. [29] who observed RVFV dissemination in *Cx. pipiens* one day post infection. Detailed data for RVFV infections in *Aedes* species are less complete, but Romoser [28] recorded that 87.5% of *Ae. mcintoshi* Huang mosquitoes infected with RVFV (Kenyan strain C6/36—6/12/86) had a disseminated infection by three days post infection. The mechanism for such rapid dissemination into the hemocoel was not clear, although it reportedly occurred prior to replication in the midgut epithelium [34].

Tissue tropisms of RVFV in *Ae. aegypti* with disseminated infections were also consistent with those reported for other mosquito species. Every tissue in which we detected RVFV antigen has been reported as commonly infected in *Cx. pipiens* [29] and *Ae. mcintoshi* [28]. Further, the sporadic timing of dissemination and the rapidity with which RVFV infected other tissues is also congruent with what has been reported previously [28]–[29]. We observed virus dissemination between days three and eight post-infection for rRVF-wt, with all specimens having a disseminated infection by day 10 post infection. For *Cx. pipiens* and *Ae. mcintoshi*, the earliest dissemination was observed one and three days post-infection, respectively, and occurred throughout the observation periods, with some specimens of each species not having disseminated infections until as late as 21 days after the infectious blood meal [28]–[29]. However in this study and in those previous, dissemination indices tended to cluster either at zero or close to one, indicating that dissemination to the various mosquito tissues was rapid once the virus entered the hemocoel. Tissue tropisms between rRVF-wt and rRVF-ΔNSm were not observably different.

We frequently also observed RVFV antigen associated with tracheal cells in the ovaries (Fig. 2C). The observation of RVFV in the tracheal system is not new. Romoser et al. [35] reported that RVFV could infect the trachea and tracheoles, and provided supporting evidence that the trachea could serve as a conduit for virus dissemination between the midgut epithelium and the hemocoel. This observation has also been reported for *Ae. mcintoshi* in Kenya [36]. Tracheal conduits have also been hypothesized to facilitate virus dissemination from the midgut in dengue 2 virus (DENV-2) infections in *Ae. aegypti* [37]. In that study, DENV-2 antigen was detected in portions of the tracheal system in approximately 35% of mosquitoes between days two and seven post-infection. Presence of viral antigen in the tracheal system was primarily in the abdominal cavity, and was strongly correlated with virus dissemination from the midgut between days two and five post-infection [37]. While this phenomenon was commonly observed for the Chetumal strain of *Ae. aegypti*, DENV-2 viral antigen was only rarely associated with the trachea of infected Rexville D *Ae. aegypti*, the strain used in this study. Infection of the ovarian tissues and the potential for vertical transmission through the tracheal cells in the ovaries is not known.

In conclusion, we have provided histological and virological evidence for the reduction in infection, dissemination, and transmission rates of RVFV lacking the NSm gene. Deletion of NSm results in the reduced ability of RVFV to replicate in and disseminate from the midgut epithelial cells. This report together with the report by Crabtree et al. [23] comprise the first description of a functional role for NSm in the vector competence of mosquitoes for RVFV.

## References

[1] Meegan JM (1979) The Rift Valley fever epizootic in Egypt 1977–78. 1. Description of the epizzotic and virological studies. Trans R Soc Trop Med Hyg 73: 618–623. 10.1016/0035-9203(79)90004-x 538803

[2] McIntosh BM, Russell D, Dos Santos I, Gear JH (1980) Rift Valley fever in humans in South Africa. S Afr Med J 58: 803–806. 7192434

[3] Madani TA, Al-Mazrou YY, Al-Jeffri MH, Mishkas AA, Al-Rabeah AM, et al (2003) Rift Valley fever epidemic in Saudi Arabia: epidemiological, clinical, and laboratory characteristics. Clin Infect Dis 37: 1084–1092. 14523773 10.1086/378747

[4] Bird BH, Ksiazek TG, Nichol ST, Maclachlan NJ (2009) Rift Valley fever virus. J Am Vet Med Assoc 234: 883–893. 19335238 10.2460/javma.234.7.883

[5] Ali H, Ali AA, Atta MS, Cepica A (2012) Common, emerging, vector-borne and infrequent abortogenic virus infections of cattle. Transbound Emerg Dis 59: 11–25. 21733134 10.1111/j.1865-1682.2011.01240.x

[6] Kasari TR, Carr DA, Lynn TV, Weaver JT (2008) Evaluation of pathways for release of Rift Valley fever virus into domestic ruminant livestock, ruminant wildlife, and human populations in the continental United States. J Am Vet Med Assoc 232: 514–529. 18279085 10.2460/javma.232.4.514

[7] USDA-APHIS CDC (2010) National Select Agent Registry. Available at: www.selectagents.gov. Accessed 16 October 2012.

[8] Centers for Disease Control (2000) Outbreak of Rift Valley fever–Yemen, August–October 2000. MMWR Morb Mortal Wkly Rep 49: 1065–1066. 11186611

[9] Al-Afaleq AI, Hussein MF (2011) The status of Rift Valley fever in animals in Saudi Arabia: a mini review. Vector Borne Zoonotic Dis 11: 1513–1520. 21923257 10.1089/vbz.2010.0245

[10] Bouloy M, Janzen C, Vialat P, Khun H, Pavlovic J, et al (2001) Genetic evidence for an interferon-antagonistic function of Rift Valley fever virus nonstructural protein NSs. J Virol 75: 1371–1377. 11152510 10.1128/JVI.75.3.1371-1377.2001 PMC114043

[11] Billecocq A, Spiegel M, Vialat P, Kohl A, Weber F, et al (2004) NSs protein of Rift Valley fever virus blocks interferon production by inhibiting host gene transcription. J Virol 78: 9798–9806. 15331713 10.1128/JVI.78.18.9798-9806.2004 PMC514997

[12] LeMay N, Dubaele S, Proietti De Santis L, Billecocq A, Bouloy M, et al (2004) TFIIH transcription factor, a target for the Rift Valley fever hemorrhagic fever virus. Cell 116: 541–550. 14980221 10.1016/s0092-8674(04)00132-1

[13] Won S, Ikegami T, Peters CJ, Makino S (2007) NSm protein of Rift Valley fever virus suppresses virus-induced apoptosis. J Virol 81: 13335–13345. 17913816 10.1128/JVI.01238-07 PMC2168885

[14] Gerrard SR, Bird BH, Albarino CG, Nichol ST (2007) The NSm proteins of Rift Valley fever virus are dispensable for maturation, replication, and infection. Virol 359: 459–465. 10.1016/j.virol.2006.09.035 PMC2364454 17070883

[15] Ikegami T, Won S, Peters CJ, Makino S (2006) Rescue of infectious Rift Valley fever virus entirely from cDNA, analysis of virus lacking the NSs gene and expression of a foreign gene. J Virol 80: 2933–2940. 16501102 10.1128/JVI.80.6.2933-2940.2006 PMC1395455

[16] Muller R, Saluzzo J-F, Lopez N, Dreier T, Turell M, Smith J, Bouloy M (1995) Characterization of clone 13, a naturally attenuated a virulent isolate of Rift Valley fever virus, which is altered in the small segment. Am J Trop Med Hyg 53: 405–411. 7485695 10.4269/ajtmh.1995.53.405

[17] Hunter P, Erasmus BJ, Vorster JH (2002) Teratogenicity of a mutagenized Rift Valley fever virus (MVP12) in sheep. Onderstepoort J Vet Re 69: 95–98. 12092782

[18] Botros B, Omar A, Elian K, Mohamed G, Soliman A, et al (2006) Adverse response of non-indigenous cattle of European breeds to live attenuated Smithburn Rift Valley fever vaccine. J Med Virol 78: 787–791. 16628582 10.1002/jmv.20624

[19] Bird BH, Albarino CG, Hartman AL, Erickson BR, Ksiazek TG, et al (2008) Rift Valley fever virus lacking the NSs and NSm genes is highly attenuated, confers protective immunity from virulent virus challenge, and allows for differential identification of infected and vaccinated animals. J Virol 82: 2681–2691. 18199647 10.1128/JVI.02501-07 PMC2258974

[20] Bird BH, Maartens H, Campbell S, Erasmus BJ, Erickson BR, et al (2011) Rift Valley fever virus vaccine lacking the NSs and NSm genes is safe, nonteratogenic, and confers protection from viremia, pyrexia, and abortion following challenge in adult and pregnant sheep. J Virol 85: 12901–12909. 21976656 10.1128/JVI.06046-11 PMC3233145

[21] National Select Agent Registry (2013) Select Agent and Toxin Exclusions. Available at: http://www.selectagents.gov/Select%20Agents%20and%20Toxins%20Exclusions.html. Accessed 30 July 2013.

[22] Bird BH, Albarino CG, Nichol S (2007) Rift Valley fever virus lacking NSm proteins retains high virulence in vivo and may provide a model of human delayed onset neurologic disease. Virol 362: 10–15. 10.1016/j.virol.2007.01.046 17412386

[23] Crabtree MB, Crockett RJK, Bird BH, Nichol ST, Erickson BR, et al (2012) Infection and Transmission of Rift Valley Fever Viruses Lacking the NSs and/or NSm Genes in Mosquitoes: Potential Role for NSm in Mosquito Infection. PLoS NTD 6: e1639. 10.1371/journal.pntd.0001639 PMC3341344 22563517

[24] Miller BR, Mitchell CJ (1991) Genetic selection of a flavivirus-refractory strain of the Yellow fever mosquito Aedes aegypti. Am J Trop Med Hyg 45: 399–407. 1659238 10.4269/ajtmh.1991.45.399

[25] Miller BR, Mitchell CJ, Ballinger ME (1989) Replication, tissue tropisms and transmission of yellow fever virus in Aedes albopictus. Trans R Soc Trop Med Hyg 83: 252–255. 2609379 10.1016/0035-9203(89)90667-6

[26] Kading R, Crabtree M, Miller B (2013) Inactivation of infectious virus and serological detection of virus antigen in Rift Valley fever virus-exposed mosquitoes fixed with paraformaldehyde. J Virol Methods 189: 184–188. 23391826 10.1016/j.jviromet.2013.01.014

[27] Kent RJ, Crabtree MB, Miller BR (2010) Transmission of West Nile virus by *Culex quinquefasciatus* Say infected with Culex flavivirus Izabal. PLoS Negl Trop Dis 4: e671. 20454569 10.1371/journal.pntd.0000671 PMC2864301

[28] Romoser WS (1989) Studies of infection and dissemination of Rift Valley fever virus in mosquitoes. Annual Report. Ohio University. Available at: http://www.dtic.mil/dtic/tr/fulltext/u2/a210219.pdf. Accessed 19 Dec 2012.

[29] Romoser WS, Faran ME, Bailey CL, Lerdthusnee K (1992) An immunocytochemical study of the distribution of Rift Valley fever virus in the mosquito *Culex pipiens* . Am J Trop Med Hyg 46: 489–501. 1575297 10.4269/ajtmh.1992.46.489

[30] Won S, Ikegami T, Peters CJ, Makino S (2006) NSm and 78-Kilodalton Proteins of Rift Valley Fever Virus Are Nonessential for Viral Replication in Cell Culture. J Virol 80: 8274–8278. 16873285 10.1128/JVI.00476-06 PMC1563821

[31] Engdahl C, Naslund J, Lindgren L, Ahlm C, Bucht G (2012) The Rift Valley fever virus protein NSm and putative cellular protein interactions. Virol J 9: 139. 22838834 10.1186/1743-422X-9-139 PMC3439357

[32] Keene KM, Foy BD, Sanchez-Vargas I, Beaty BJ, Blair CD, et al (2004) RNA interference acts as a natural antiviral response to O'nyong-nyong virus (Alphavirus: Togaviridae) infection of *Anopheles gambiae* . PNAS 101: 17240–17245. 15583140 10.1073/pnas.0406983101 PMC535383

[33] Shelly S, Lukinova N, Bambina S, Berman A, Cherry S (2009) Autophagy is an essential component of *Drosophila* immunity against vesicular stomatitis virus. Immunity 30: 588–598. 19362021 10.1016/j.immuni.2009.02.009 PMC2754303

[34] Faran ME, Romoser WS, Routier RG, Bailey CL (1988) The distribution of Rift Valley Fever virus in the mosquito *Culex pipiens* as revealed by viral titration of dissected organs and tissues. Am J Trop Med Hyg 39: 206–213. 3407841 10.4269/ajtmh.1988.39.206

[35] Romoser WS, Turell MJ, Lerdthusnee K, Neira M, Dohm D, Ludwig G, Wasielowski L (2005) Pathogenesis of Rift Valley fever virus in mosquitoes—tracheal conduits and the basal lamina as an extra-cellular barrier. Arch Virol Suppl 19: 89–100. 10.1007/3-211-29981-5_8 16355869

[36] Romoser WS, Oviedo MN, Lerdthusnee K, Patrican LA, Turell MJ, et al (2011) Rift Valley fever virus-infected mosquito ova and associated pathology: possible implications for endemic maintenance. Res Rep Trop Med 2: 121–127. 10.2147/RRTM.S13947 PMC6415639 30881185

[37] Salazar MI, Richardson JH, Sanchez-Vargas I, Olson KE, Beaty BJ (2007) Dengue virus type 2: replication and tropisms in orally infected *Aedes aegypti* mosquitoes. BMC Microbiol 7: 9. 17263893 10.1186/1471-2180-7-9 PMC1797809

